# Effects of Lipophilic Extract of *Viscum album* L. and Oleanolic Acid on Migratory Activity of NIH/3T3 Fibroblasts and on HaCat Keratinocytes

**DOI:** 10.1155/2013/718105

**Published:** 2013-11-26

**Authors:** R. Kuonen, U. Weissenstein, K. Urech, M. Kunz, K. Hostanska, M. Estko, P. Heusser, S. Baumgartner

**Affiliations:** ^1^Society for Cancer Research, Hiscia Institute, 4144 Arlesheim, Switzerland; ^2^Institute for Complementary Medicine, University Hospital Zurich, 8091 Zurich, Switzerland; ^3^Witten/Herdecke University, 58448 Witten, Germany; ^4^Institute of Integrative Medicine, Witten/Herdecke University, 58448 Witten, Germany; ^5^Institute of Complementary Medicine KIKOM, University of Bern, 3010 Bern, Switzerland

## Abstract

*Viscum album* L. lipophilic extract (VALE) contains pharmacologically active pentacyclic triterpenes that are known to exhibit immunomodulatory, antitumor, and wound healing activity. Preliminary clinical observations indicate that VALE was able to influence cutaneous wound healing *in vivo*. The objective of this study was to investigate wound closure related properties of VALE *in vitro*. As measured in a wound healing assay, VALE and its predominant triterpene oleanolic acid (OA) significantly and dose dependently promoted the migration of NIH/3T3 fibroblasts *in vitro*, thereby leading to an enhanced wound closure. Compared to the negative control, maximal stimulation by 26.1% and 26.2%, respectively, was attained with 10 *μ*g/mL VALE and 1 *μ*g/mL OA. Stimulation of proliferation in NIH/3T3 fibroblasts by VALE and OA could be excluded. At higher concentrations both substances affected proliferation and viability of NIH/3T3 fibroblasts and HaCat keratinocytes. In the toxic range of concentrations of VALE and OA, migration of NIH/3T3 fibroblasts was suppressed. The extent of the stimulatory effect on cell migration of VALE quite closely corresponded to the effect expected by the concentrations of OA contained in the crude extract VALE. These data support the casual observation that *Viscum album* L. lipophilic extract might modulate wound healing related processes *in vivo*.

## 1. Introduction

Pentacyclic triterpenes like OA, betulinic acid, and ursolic acid are lipophilic compounds of mistletoe with promising pharmacological activities [1–3]. Several properties are described for OA and betulinic acid including antitumor [4–6], anti-inflammatory [7], anti-HIV [8, 9], and immunomodulatory activities [10]. Additionally they exert remarkable wound healing properties [11] and it has been shown that plant extracts containing pentacyclic triterpenes are effective in the topical treatment of actinic keratosis [12]. In a prospective case series study, Kunz et al. [13] used an ointment containing *Viscum album* lipophilic extract (VALE unguentum) for the topical treatment of basal cell carcinoma (BCC). Their clinical findings include promotion of wound healing and antitumoral properties of the lipophilic mistletoe extract. A primary hemostatic effect of the treatment followed by wound closure and partial or complete remission of BCC was observed.

Cutaneous wound healing is a complex self-limiting process characterized by a well coordinated, progressive sequence of events and is structured with regard to both time and space [14]. The acute wound healing process proceeds in three partly overlapping phases: hemostasis and inflammation, granulation tissue formation and reepithelialization, and wound remodeling, and many different cell types are involved, for example, fibroblasts and keratinocytes [15, 16]. As fibroblasts are responsible for initiating angiogenesis, epithelialization, collagen formation, and synthesis of extracellular matrix proteins [15, 17] an important step of the second phase consists in activation of fibroblast migration into the wounded area.

In our study we used a well-established *in vitro* assay to mimic the migration of mouse NIH/3T3 fibroblasts to an artificial wound with the objective to evaluate the pharmacological effects of VALE and OA on wound healing and we tested their effects on viability and proliferation of fibroblasts and keratinocytes.

## 2. Material and Methods

### 2.1. Cell Lines and Cell Culture Conditions

NIH/3T3 mouse fibroblasts (ATCC, Rockville, MD, USA) were cultured in RPMI-1640 medium supplemented with 5% heat-inactivated fetal calf serum (FCS), 2 mM L-Glutamine, and 1% penicillin-streptomycin. HaCat human adult keratinocytes (Institute of Pathology, University Hospital Basel, Switzerland) were cultured in Dulbecco's Modified Eagle's medium (DMEM low glucose) supplemented with 10% heat-inactivated FCS, 2 mM L-Glutamine, and 1% penicillin-streptomycin at 37°C in a humidified atmosphere containing 5% CO_2_. For the experiments, cells were collected from subconfluent monolayers using trypsin/EDTA and further cultured in medium containing 1% FCS. All cell culture reagents were obtained from Sigma (Buchs, Switzerland).

### 2.2. Reagents and Extracts

Oleanolic acid (OA, purity > 98%) and dimethyl sulfoxide (DMSO) were purchased from MP Biomedicals Europe and from Sigma (Buchs, Switzerland), respectively. *Viscum album* L. lipophilic extract (VALE), containing 10% of OA, was obtained by ultracritical CO_2_ extraction and was kindly provided by Hiscia Institute, Verein für Krebsforschung Arlesheim, Switzerland. OA and VALE were dissolved in DMSO. Final concentrations of DMSO in all cell culture assays never surpassed the concentration of 1%.

### 2.3. WST-1 Cell Viability Assay

The viability of NIH/3T3 and HaCat cells was measured by the use of a colorimetric WST-1 assay based on the cleavage of the tetrazolium salt WST-1 by viable cells, according to the supplier's instructions (Roche Diagnostica, Rotkreuz, Switzerland). In brief, cells were seeded into 96-well microtiter plates at a density of 2.5–5 × 10^3^cells per well. NIH/3T3 cells were treated with either VALE (25–2000 *μ*g/mL) or OA (6.13–100 *μ*g/mL) for 24 h and 48 h and HaCat cells were treated with either VALE (100–1600 *μ*g/mL) or OA (1.25–80 *μ*g/mL) for 24 h and 48 h, followed by incubation with WST-1 for 4 h. Absorbance at 450 nm and 650 nm against a background control was measured in a multi-well plate reader (Labsystems Multiscan RC, Helsinki, Finland). The effect of vehicle DMSO on the cell viability was tested in parallel.

Three independent experiments performed in triplicates were evaluated. Cell growth inhibition was calculated as follows: inhibition in % = 100 − (absorbance of treated cells/absorbance of untreated control ∗ 100).

### 2.4. *In Vitro* Wound Healing Assay

The effect of VALE and OA on wound closure was investigated using a CytoSelect 24-well wound healing assay (Cell Biolabs, Inc., San Diego, USA).

3 × 10^4^ NIH/3T3 fibroblasts in DMEM (1% FCS) were seeded into the inserts of a CytoSelect 24-well plate and cultured overnight to allow adhering and reaching a 60–80% confluence. The inserts were carefully removed, leaving a defined and precise 0.9 mm wound field. The medium was aspirated and cells were further incubated for 24 h with either VALE (0.1–2000 *μ*g/mL) or oleanolic acid (0.1–50 *μ*g/mL) in DMEM medium supplemented with 1% FCS. For the determination of cell migration to the wound field, cells were stained according to manufacturer's instructions.

To define confluent area (100%), NIH/3T3 cells were seeded into wells without created wounded area, allowed adhering and reaching confluence for 24 h, and subsequently stained.

DMEM with 5% FCS was used as positive control and medium with 1% FCS as negative control.

For visualization microscopic images focused on the center of the wound were photographed and analyzed using CellD imaging software [18]. The wound healing assay was repeated 7 times.

For the statistical analysis, all data were standardized to the density of confluent cells (100%) and expressed as percentage of cells in wounded area (%) = (test compound/confluent area) × 100. 

### 2.5. BrdU Proliferation Assay

Cell proliferation was evaluated by 5-bromo-2-deoxyuridine (BrdU) incorporation using a colorimetric BrdU Cell Proliferation ELISA (Roche Applied Science, Indianapolis, IN, USA) according to manufacturer's instructions. Briefly, NIH/3T3 cells were seeded at a density of 5 × 10^3^/well into a 96-well microtiter plate and allowed to adhere for 4–6 h in DMEM medium supplemented with 1% FCS. Then cells were cultured in DMEM in the presence of VALE (0.01–100 *μ*g/mL) or OA (0.001–10 *μ*g/mL) or in the absence of tested compounds for 24 h and 48 h. During the final 24 h of culture, 10 *μ*M BrdU was added. After DNA denaturation and incubation with anti-BrdU-POD antibody the absorbance at 450/690 nm was measured using a multiwell plate reader. Wells with unlabelled cells were set as background control and wells with culture medium alone were set as blank. Three independent experiments performed in triplicates were evaluated.

### 2.6. Statistics

Viability data from the WST-1 test were assessed with descriptive statistics (calculation of mean and standard error (SE)). Data of the wound healing and proliferation assays were analyzed with analysis of variance (ANOVA) using the software Statistica 4.0 (Statsoft, Tulsa, USA). Global significance was assessed with the *F*-test. Only if the preceding *F*-test was significant pairwise comparisons with the LSD-test was carried out (protected Fisher's LSD-test). *P* values less than 0.05 were considered to be statistically significant.

## 3. Results

### 3.1. Effects of VALE and OA on Cell Viability

As cytotoxic effects can influence results of the functional wound healing assay, the effect of VALE and OA on cell viability after 24 h and 48 h treatment was measured by determining metabolic activity using the WST-1 assay.

**Figure 1:**
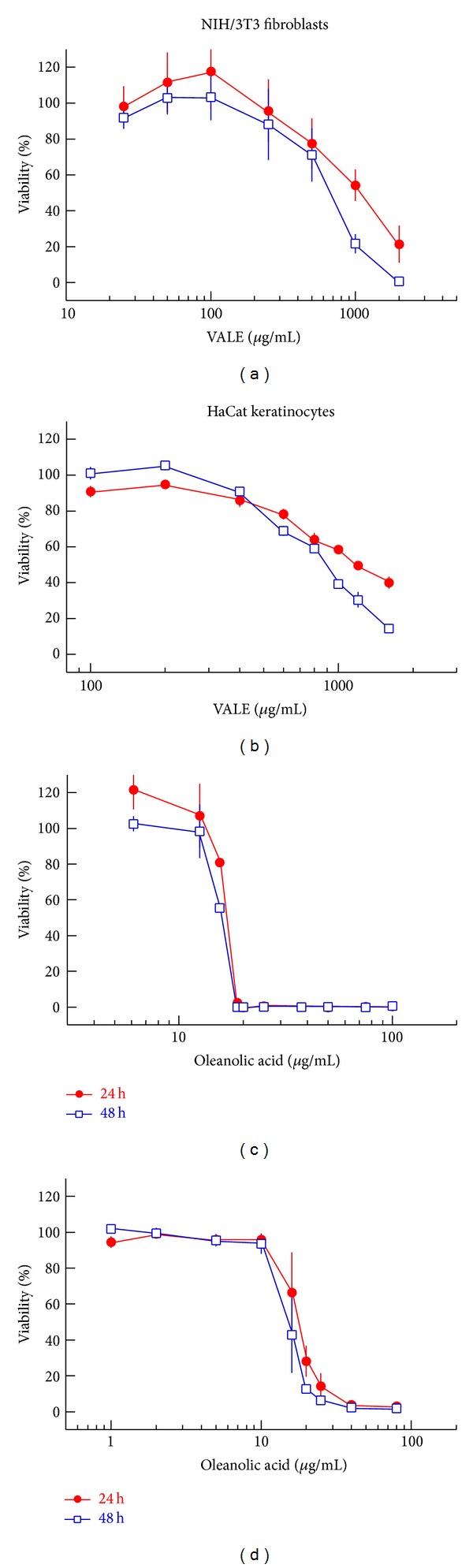
Dose-dependent cytotoxic effect of VALE on (a) NIH/3T3 and (b) HaCat cells and of OA on (c) NIH/3T3 and (d) HaCat cells after 24 h and 48 h of incubation. Mean values ± SE of three experiments are expressed in percentage of cell viability compared to the untreated control.

VALE investigated at concentrations from 25 to 2000 *μ*g/mL in NIH/3T3 cells and from 100 to 1600 *μ*g/mL in HaCat cells decreased the viability of both cell lines in a dose-dependent manner (Figures 1(a) and 1(b)). NIH/3T3 cells were slightly more sensitive to VALE than HaCat cells. 

OA displayed a steep dose-dependent cytotoxic effect on both NIH/3T3 and HaCat cells within a small dose range (Figures 1(c) and 1(d)). Both cell lines exhibited an almost complete abrogation of cell viability by concentrations between 18 and 100 *μ*g/mL OA after 48 h of treatment. Concentrations of VALE <100 *μ*g/mL and OA <12.5 *μ*g/mL were nontoxic for NIH/3T3 fibroblasts, and VALE <200 *μ*g/mL and OA < 10 *μ*g/mL were nontoxic for HaCat keratinocytes (cell viability >90%).

The amount of DMSO contained in the tested samples was used as control to determine the effect of DMSO on untreated cells. No toxic effect was observed below 1% DMSO (data not shown).

### 3.2. Effect of VALE and OA on Migratory Activity and Cell Growth of NIH/3T3 Fibroblasts

**Figure 2:**
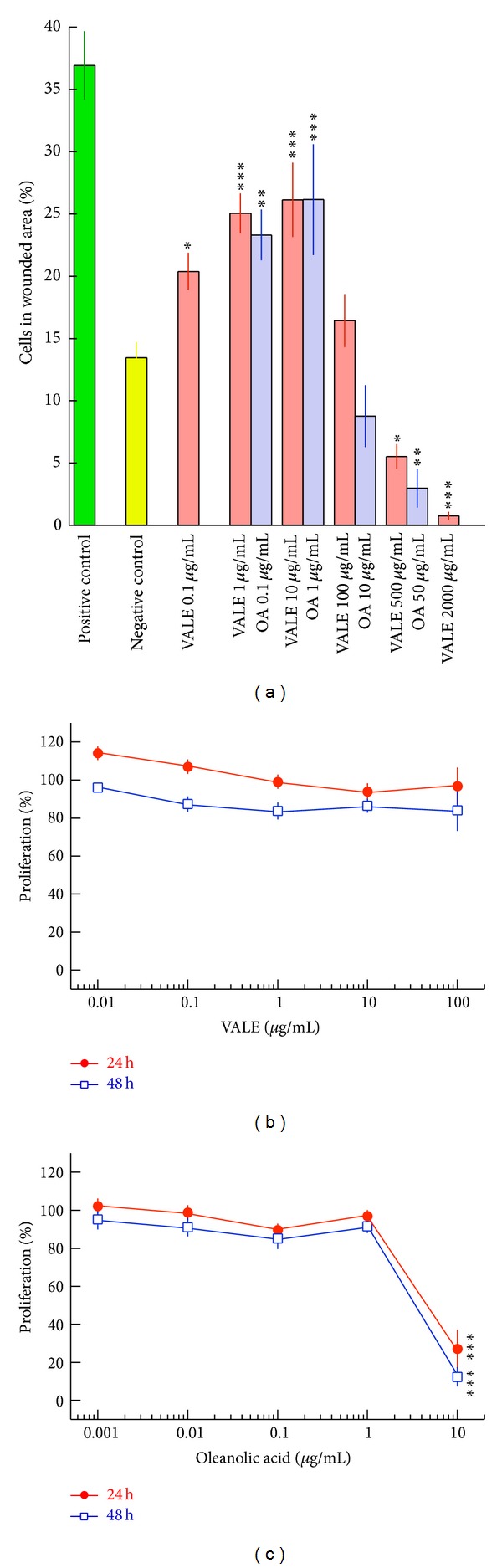
(a) Effect of VALE and OA on wound closure of NIH/3T3 fibroblasts expressed in % of cells migrated to the wounded area. As positive control DMEM with 5% FCS and as negative control untreated samples (1% FCS) were used. Density of confluent cells without created wound was set as 100% wound closure. (b), (c) Effect of VALE and OA on the proliferation of NIH/3T3 fibroblasts was expressed as proliferation of cells compared to the untreated control. Results are expressed as means ± SE of 7 independent wound healing or 3 proliferation experiments, respectively. Significance levels are given compared to the negative control (*P < 0.05, **P < 0.01, ***P < 0.001, LSD-test).

**Figure 3:**
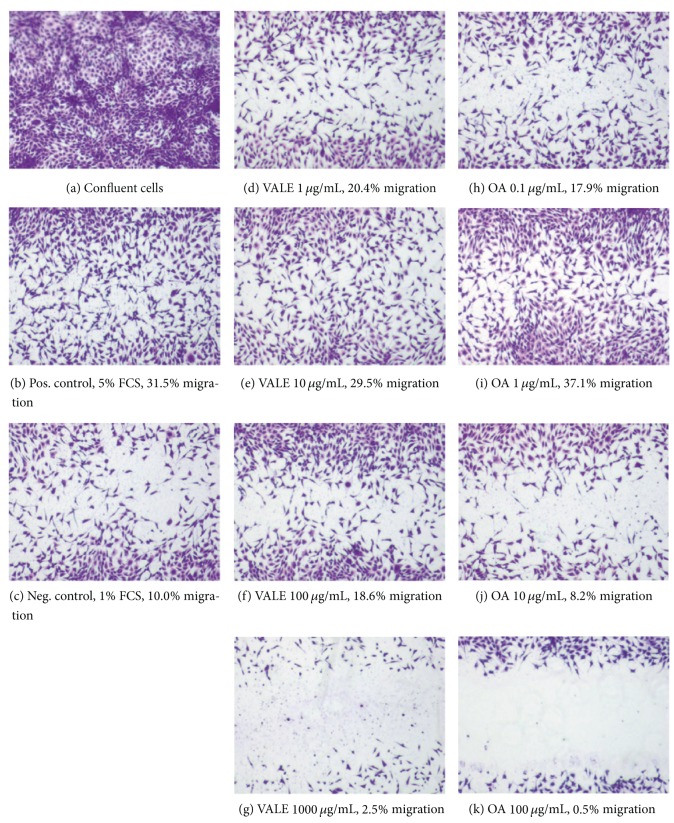
Representative microphotographs of one experiment, illustrating the dose-dependent effect of VALE (d)–(g) and OA (h)–(k) on NIH/3T3 fibroblast migration 24 h after monolayer wounding. Data were standardized to the density of confluent cells (100%) (a) and expressed as percentage of cells in wounded area (% migration) = (test compound/confluent area) × 100. Samples treated with 5% FCS were set as positive control (b) and untreated samples (1% FCS) were set as negative control (c).

The effect of VALE and OA on the migratory activity of NIH/3T3 fibroblasts was evaluated using a wound healing assay. Results are expressed as percentage of cells in wounded area (Figure 2(a)). 36.9% of NIH/3T3 positive control cells (5% FCS) migrated to the wounded area in contrast with 13.5% in the untreated negative control (1% FCS). Compared to the negative control, VALE at concentrations between 0.1 and 10 *μ*g/mL and OA at concentrations of 0.1 *μ*g/mL and 1.0 *μ*g/mL significantly promoted the migration of NIH/3T3 cells thereby leading to an enhanced wound closure. VALE at concentrations from 500 to 2000 *μ*g/mL and 50 *μ*g/mL of OA significantly inhibited the wound closure (Figure 2(a)). Maximal stimulation of NIH/3T3 cell migration by VALE and OA was found at concentrations of 10 *μ*g/mL (26.1%, *P* < 0.001) and 1 *μ*g/mL (26.2%, *P* < 0.001), respectively. The dose-dependent wound healing effect of VALE and OA is illustrated in Figure 3 showing different extents of wound closure in the original microphotographs of one representative experiment.

The proliferative response of NIH/3T3 cells on VALE and OA was assessed by quantitative analysis of the percentage of cells staining positive for BrdU incorporation. The levels of proliferation found in response to VALE and OA were compared to those of the untreated control (1% FCS).

No proliferation stimulating effect could be found with both substances for the cells at any concentration (Figures 2(b) and 2(c)). A strong and statistically significant inhibition of proliferation by about 80% was found with OA at a concentration of 10 *μ*g/mL.

## 4. Discussion

An important finding of our *in vitro* study was the stimulatory effect of VALE and OA on the migratory activity of NIH/3T3 fibroblasts leading to an enhanced wound closure. This effect resulted from stimulation of fibroblasts motility rather than from their mitosis.

Several *in vivo* studies show wound healing activities of triterpenes. Metelmann et al. reported remarkable advantage in promoting wound healing in superficial wounds treated with triterpenes compared to a moist wound dressing [11]. Severe necrotizing herpes zoster of an immunosuppressed patient was successfully treated with an emulsion of the pentacyclic triterpene betulin without causing any side effects [19]. Oleanolic acid isolated from *Anredera diffusa* induced an enhanced wound healing activity in mice [20]. In experimental animal models the activity of the triterpene glycoside asiaticoside, isolated from *Centella asiatica*, improved and facilitated normal as well as delayed-type wound healing and related properties like increase in hydroxyproline, tensile strength, and collagen content and promoted epithelialization and angiogenesis [21]. Kunz et al. [13] observed in a prospective case series study wound healing promoting and anti-tumoral effects by topical treatment of BCC with VALE unguentum and more specifically an achievement of hemostasis in bleeding tumor wounds and after a prolonged treatment period a wound reepithelialization with a thin epithelial layer (personal communication).

In the present study we investigated the effect of lipophilic mistletoe extract on cutaneous wound healing *in vitro*. NIH/3T3 fibroblast cell cultures have been widely used as a model for testing wound healing activity *in vitro *[22, 23] because cell migration, proliferation, and collagen production of fibroblasts play an essential role in cutaneous wound healing. Since growth inhibiting and apoptosis-inducing properties of lipophilic extract from *Viscum album* L. and of its predominant triterpene oleanolic acid have been demonstrated [2], initially the determination of the nontoxic range of VALE and OA was crucial to choose appropriate concentrations when performing the functional wound healing assay. Cell survival was estimated after 24 h and 48 h of treatment using the WST-1 assay. Cells with a viability > 90% of the untreated control were considered to be unaffected. Our results demonstrated that concentrations of VALE < 100 *μ*g/mL and of OA < 12.5 *μ*g/mL were nontoxic for NIH/3T3 fibroblasts. HaCat keratinocytes were slightly less sensitive and concentrations of VALE < 200 *μ*g/mL and of OA < 10 *μ*g/mL were revealed to be nontoxic for this cell type. We thus observed a comparable effect of VALE and OA on the viability of fibroblasts and keratinocytes, cell types whose proliferation, migration, and complex interactions play a primary role in wound healing [24]. Parallel experiments revealed similar dose response curves for NIH/3T3 cell viability after treatment with betulinic acid with nontoxic concentrations <2.5–5.0 *μ*g/mL (data not shown).

Fibroblast migration is one of the essential steps during the proliferative phase of wound healing and during wound closure. The effect of VALE and OA on the migration of NIH/3T3 fibroblasts was investigated using the CytoSelect 24-well wound healing assay. Former experiments performed under similar cell culture and assay conditions illustrate the reliability of the assay to study the influence of wound healing compounds like Bepanthen Plus (Bayer Schweiz AG) on NIH/3T3 fibroblast migration (see supplementary material available online at http://dx.doi.org/10.1155/2013/718105 ). We observed a dose-dependent stimulation of migration at nontoxic doses of VALE (0.1–10 *μ*g/mL) and of OA (0.1–1 *μ*g/mL) and an inhibition of migration at higher doses of VALE (500–2000 *μ*g/mL) and of OA (50 *μ*g/mL). This inhibitory effect on fibroblast migration corresponds to our results of the cytotoxic properties of VALE and OA in NIH/3T3 fibroblasts. With a proportion of 10%, OA is the most prominent triterpene found in VALE. The strongest stimulation of migration was found with 10 *μ*g/mL of VALE and its corresponding concentration of 1 *μ*g/mL of OA. For this reason we hypothesize that the main active component in VALE responsible for the stimulatory effect on NIH/3T3 fibroblast migration was OA.

Cell migration is not the only component which contributes to wound closure. Cell proliferation has also to be taken into account. We investigated a possible induction of NIH/3T3 fibroblast proliferation by VALE and OA as the wound healing assay used does not allow a clear distinction between migration and proliferation. The results of the BrdU incorporation assay revealed no mitogenic effect of VALE and OA on NIH/3T3 fibroblasts under the experimental conditions used. Enhanced NIH/3T3 cell numbers in an artificial wound by triterpene containing birch bark extract as well as isolated triterpenes in a concentration-dependent manner were also reported by Naumann et al. [25]. In their study, fibroblast proliferation as well as cell migration was influenced. Additionally, HaCat cells treated with the birch bark extract revealed a time-dependent increase of COX-2 mRNA, the key enzyme for the prostaglandin E2 production. Cells use different signaling pathways for migration from those for proliferation. For example, migratory effects of members of heparin-binding fibroblast growth factor family (FGFs) are clearly discriminated from proliferative effects [26]. The mechanisms and signaling pathways responsible for the migratory effect of VALE and OA on NIH/3T3 fibroblasts are unclear at present and need further investigation.

## 5. Conclusions

Clinical observations indicate a relevant contribution of *Viscum album* lipophilic extract (VALE) to wound healing *in vivo*, thus highlighting the therapeutic potential of the lipophilic fraction of mistletoe. Our *in vitro* results demonstrate that VALE and oleanolic acid stimulated the migration of NIH/3T3 fibroblasts *in vitro*, which led to an enhanced wound closure. These findings are a first step in the process of investigating the underlying mechanisms of the effects of VALE on wound healing.

## Supplementary Material

In Figure 1S, microphotographs of two experiments illustrating the effect of the wound healing compound Bepanthen ®Plus (Bayer Schweiz AG, dexpanthenol 50 mg, chlorhexidine digluconate 5 mg per 1 ml) on NIH/3T3 fibroblast migration are shown. Using the CytoSelectTM 24-well wound healing assay (Cell Biolabs, Inc.,San Diego, USA), these experiments were performed under similar cell culture and assay conditions as the experiments with VALE and OA.
