# Supplementary material for: Effects of Lipophilic Extract of Viscum album L. and Oleanolic Acid on Migratory Activity of NIH/3T3 Fibroblasts and on HaCat Keratinocytes
Source: Evid Based Complement Alternat Med. 2013 Nov 26;2013:718105. doi: 10.1155/2013/718105 (PMC3860131; doi:10.1155/2013/718105)
Supplement: Supplementary file 1 — In Figure 1S, microphotographs of two experiments illustrating the effect of the wound healing compound Bepanthen ®Plus (Bayer Schweiz AG, dexpanthenol 50 mg, chlorhexidine digluconate 5 mg per 1 ml) on NIH/3T3 fibroblast migration are shown. Using the CytoSelectTM 24-well wound healing assay (Cell Biolabs, Inc.,San Diego, USA), these experiments were performed under similar cell culture and assay conditions as the experiments with VALE and OA. [file 718105.f1.pdf]

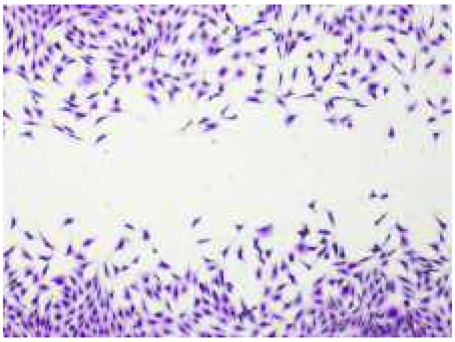

1A: Control 2% FCS 3.2%

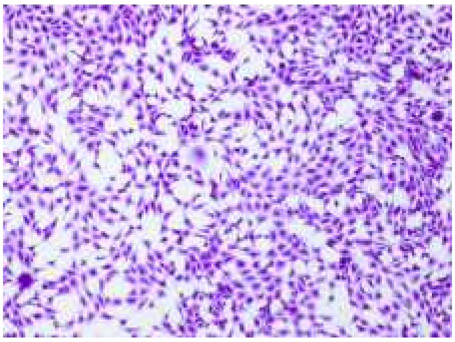

1B: Confluent area 35.3%

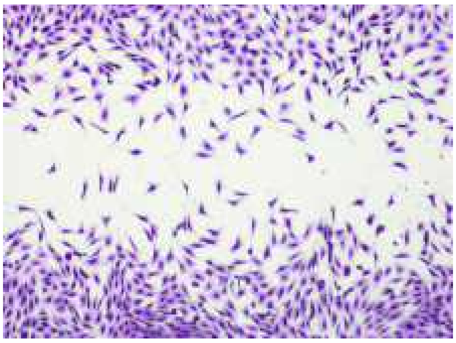

1C: Pos. control 5% FCS 14.2%

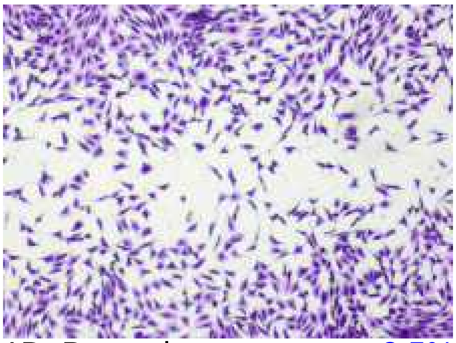

1D: Bepanthen 8.5%

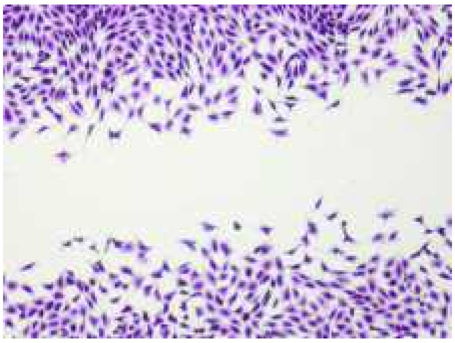

2A: Control 2% FCS 4.5%

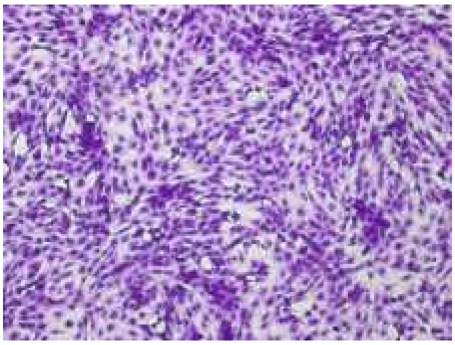

2B: Confluent area 42.7%

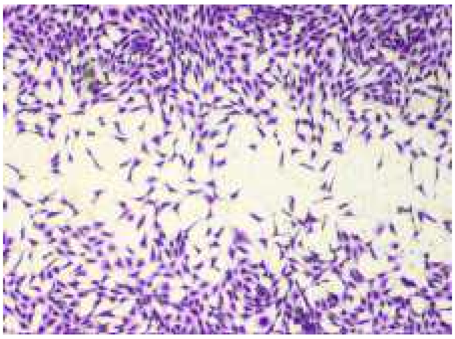

2C: Pos. control 5% FCS 26.3%

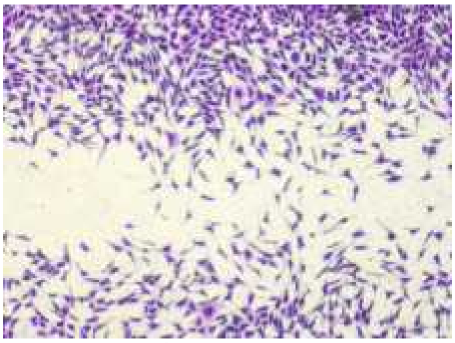

2D: Bepanthen 21.1%

Figure: Representative microphotographs of two experiments (1 and 2, kindly provided by K. Hostanska), illustrating the effect of Bepanthen® Plus (Bayer, dexpanthenol 50 mg, chlorhexidine digluconate 5 mg per 1 ml) on NIH/3T3 fibroblast migration 24 h after monolayer wounding. Data were standardized to the density of confluent cells (100 %) and expressed as percentage of cells in wounded area (% migration) = (test compound / confluent area) x 100. (1,2 A) negative control (2 % FCS), (1,2 B) confluent area, (1,2 C) positive control (5 % FCS), (1,2 D) Bepanthen (0.5 µg/ml dexpanthenol)
